# Supplementary material for: Complexome profiling of the Chlamydomonas psb28 mutant reveals TEF5 as an early PSII assembly factor
Source: Plant Cell. 2025 Mar 17;37(6):koaf055. doi: 10.1093/plcell/koaf055 (PMC12164590; doi:10.1093/plcell/koaf055)
Supplement: koaf055_Supplementary_Data [file koaf055_supplementary_data.zip › psb28_interactive.zip!/plots/intensity/qualityPlots/peptides/PRX1_Cre06.g257601.t1.2.html]

### Cre06.g257601.t1.2

Trivial name: PRX1  
SeqCoverage [median]: 24.70  
Mass: 25.96 kDa  
  
 

| Sequence | MissedCleavage | Mass [Da] | wtIntensity | muIntensity | Score |
| --- | --- | --- | --- | --- | --- |
| AQAVFDQEFQEITLSK | 0 | 1852.92 | 798.44 | 0.00 | 136.30 |
| AYGVLTEDGISLR | 0 | 1392.72 | 8433.55 | 26614.65 | 140.17 |
| EGVVQHATINNLAFGR | 0 | 1724.90 | 0.00 | 2276.78 | 38.51 |
| EISKAYGVLTEDGISLR | 1 | 1849.98 | 0.00 | 2523.23 | 41.87 |
| GSKEYFSAV | 1 | 986.47 | 1418.62 | 5610.98 | 99.31 |
|  |  |  | 10650.6 | 37025.6 |  |
